# Investigation of Bladder Microbiota in Female Patients with Overactive Bladder Syndrome

**DOI:** 10.5152/tud.2025.24040

**Published:** 2025-03-06

**Authors:** Esra Kaya, Tayfun Sahınkanat, Murat Aral

**Affiliations:** 1 Department of Medical Microbiology, Kahramanmaras Necip Fazil City Hospital, Kahramanmaraş, Türkiye; 2 Department of Urology, Kahramanmaras Sutcu Imam University School of Medicine, Kahramanmaraş, Türkiye; 3 Department of Medical Microbiology, Ankara Etlik City Hospital, Ankara, Türkiye

**Keywords:** Anaerobe culture, bladder microbiota, expanded quantitative urinary culture, overactive bladder syndrome, urinary microbiota

## Abstract

**Objective:**

With the demonstration that the bladder is not sterile, the relationship between bladder microbiota and urinary system diseases has begun to be investigated. The aim of our study is to investigate the relationship between overactive bladder (OAB) syndrome and bladder microbiota.

**Methods:**

A total of 20 OAB syndrome patients and 20 controls were included in the study. Urine samples were taken with a transurethral catheter. We developed a modified expanded quantitative urine culture method and inoculated them into anaerobic blood culture bottles and thioglycolate medium at the bedside. The MALDI-TOF MS system was used for bacterial identification.

**Results:**

Thirty-five bacteria were identified in the patient group and 30 in the control group. As a result of culture, 16 different genera and 29 different types of bacteria were identified. Staphylococcus (25.7%) was the most common bacterial genus in the patient group, followed by Streptococcus (17.1%) and Lactobacillus (14.3%). Lactobacillus (26.7%) was the most frequently detected bacterial genus in the control group, followed by Streptococcus (13.3%) and Enterococcus (13.3%). The rate of *Staphylococcus epidermidis* in the patient group (22.9%) was found to be significantly higher than in the control group (0%) (*P* = .006). In the patient group, *Lactobacillus gasseri*’s incidence (2.9%) was found to be significantly lower than in the control group (20.0%) (*P* = .042).

**Conclusion:**

Our study shows that patients with OAB have a significantly different microbiota compared to the control group.

**Main Points**

- This is the first article investigating the women urinary microbiota in Turkey.
- In the patient group, Lactobacillus gasseri was detected less frequently than in the control group, whereas Staphylococcus epidermidis was detected more frequently.
- By including the blood culture bottle in the cultur method we modified, we managed to detect more bacteria.

## Introduction

Overactive bladder (OAB) syndrome is a symptom-based syndrome characterized by urinary urgency, with increased urine frequency (≥8/day) and nocturia (≥2/night) without any pathology or infection of the urinary system. This syndrome may or may not be accompanied by urinary incontinence.^1^ The etiology of this syndrome, which is characterized by bladder storage dysfunction, has not been fully elucidated. Although there are many medical and surgical methods for treatment, some patients do not respond to any treatment. In order to develop new treatment methods for these patients, the etiology of the disease needs to be better understood.^2^,^3^ Changes in bladder microbiota may play a role in OAB syndrome. Identifying these changes may help find new treatment methods.^4^

As standard urine culture methods have been insufficient until recently, the bladder has been accepted as sterile.^5^ The presence of urinary microbiota has been known since 2010 due to the development of 16S rRNA sequencing methods independent of culture, and culture methods which facilitate the production of difficult to produce bacteria.^6^ In addition to studies conducted with healthy volunteers, research has started on various diseases involving the urinary system.^4^,^7^,^8^

Although the bladder microbiome has been investigated with 16S rRNA sequencing methods, these have not provided clear information about the living microbiota. Therefore, the expanded quantitative urine culture (EQUC) method was developed to investigate the bladder microbiota. With this culture method, it is aimed to determine the microorganisms present in the bladder using different atmospheric conditions and prolonged incubation periods.^7^

In our study, we aimed to investigate whether there was a change in the bladder microbiota in patients with OAB syndrome compared to the control group by modifying the EQUC method according to our own conditions. Additionally, our study is the first to investigate bladder microbiota in Türkiye.

## Material and Methods

### Patients and Sample Collection

The study group was formed from female patients over 18 years old, who presented at the Urology Clinic between November 2021 and February 2022, and were diagnosed with OAB. According to the 2010 ICS definition of OAB, patients must describe a sudden urinary urgency. This is usually accompanied by increased urinary frequency and nocturia. Urinary incontinence may not be present.^1^

The control group consisted of female patients over 18 years old, who required a catheter to be placed routinely after hospitalization for reasons not involving the urinary tract, and with excluded OAB diagnosis. All the study participants had no urinary tract infection (UTI) within the last month, were not using antibiotics, were not pregnant, and had no known urological or neurological pathology.

Using the G*Power 3.1 (Faul, Erdfelder, Lang, & Buchner, 2009) program, when the power was set at 80%, the total sample number was found to be 40 (patient group n = 20, control group n = 20). Signed informed consent was obtained from all the participants before enrollment in the study.

Midstream urine culture was taken from all participants and no growth was detected, thus excluding the presence of UTI. After the presence of UTI is excluded, 20 mL urine samples were taken from the participants with the help of a transurethral catheter and sterile injector. Of the 20 mL urine collected, 8-10 mL were inoculated into an anaerobic blood culture bottle (Becton Dickinson (BD), USA) at the bedside, and 2 mL in thioglycolate medium (BD, USA). The urine remaining in the syringe was sent immediately to the Medical Microbiology Laboratory for analysis.

### Standard Urine Culture

From the midstream urine sample, 0.001 mL was planted in 5% sheep blood agar (SBA) and eosin methylene blue (EMB) medium (BD, USA). The media were incubated in an aerobic environment for 24 hours at 35-37°C. The samples with no production in the midstream urine culture were included in the study.

### Modified Expanded Quantitative Urine Culture

The EQUC method, defined by Hilt et al^7^ in articles published in 2014, was modified with the following steps for this study:

1. From the urine sample in the sterile injector, 0.1 mL was inoculated into SBA, chocolate medium, and EMB medium, then incubated for 48 hours at 35-37°C in an aerobic environment. One bacteria colony produced shows 10 cob/mL bacteria.
2. After the addition of 1 mL urine to thioglycollate medium and incubation for 24 hours at 35-37°C, 0.1 mL was taken and inoculated into SBA and chocolate agar, then incubated for 48 hours at 35-37°C in an incubator with CO_2_. One bacterial colony produced shows 10 cob/mL bacteria.
3. After the addition of 1 mL urine to thioglycollate medium and incubation for 24 hours at 35-37°C, 0.1 mL was taken and inoculated into BD BBL™ CDC Anaerobe 5% SBA (BD, USA) and then incubated for 5 days at 35-37°C with BD BBL™ GasPak™ anaerobic indicator (BD, USA) inside a gas pack.
4. After the addition of 1 mL urine to thioglycollate medium and incubation for 24 hours at 35-37°C, 0.1 mL was taken and inoculated into SBA and incubated for 48 hours at 35-37°C in an aerobic environment. One bacteria colony produced shows 10 cob/mL bacteria.
5. With a positive signal given from the anaerobic blood culture bottle, 0.1 mL was inoculated into BD BBL™ CDC Anaerobe 5% SBA (BD, USA) and then incubated for 5 days at 35-37°C with BD BBL™ GasPak™ anaerobic indicator (BD, USA) inside a gas pack. Another 0.1 mL was taken and inoculated into SBA and incubated for 5 days at 35-37°C in an aerobic environment.

Identification of all the colonies produced in the modified EQUC was made using MALDI-TOF MS (Bruker Daltonik GmbH, Leipzig, Germany).

### Ethics Committee Approval

The protocol for this research project has been approved by a suitably constituted Ethics Committee of the Institution and it conforms to the provisions of the Declaration of Helsinki. Committee of Kahramanmaraş Sütçü İmam University Non-interventional Clinical Research Ethics Committee on March 22, 2021, Decision no.: 04, Session no.: 2021/11.

## Results

A total of 40 urine specimens were examined using the modified EQUC techniques. Table 1 shows whether microorganisms were detected in the samples under different conditions. With the modified EQUC method, 35 bacteria were identified in the patient group and 30 bacteria were identified in the control group. As a result of the inoculations performed, 16 different genuses and 29 different species of bacteria were identified. The microorganisms identified in the patient and control groups are shown according to genus distribution and percentage in Table 2. No statistically significant difference was determined between the groups in respect of the distribution of bacteria genuses (*P* > .05).

**Figure 1.**
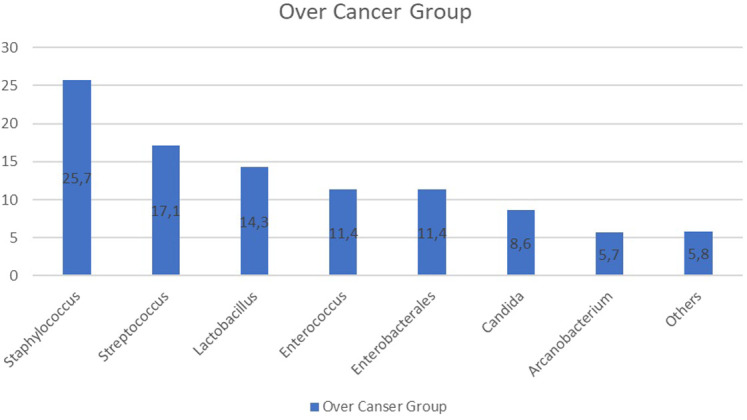
Microorganism distribution in the ovarian cancer group.

The distribution of the microorganisms identified in the patient and control groups according to species is shown in Table 3. As shown in Table 3, the most common bacterial species in the patient group was *Staphylococcus epidermidis* (n = 8, 22.9%), followed by *Streptococcus anginosus* (n = 4, 11.4%) and *Enterococcus faecalis* (n = 3, 8.6%). The anaerobic bacteria *Actinomyces urogenitalis* was identified in the patient group. The distributions of the microorganisms identified in the patient group are shown in Table 1 and Figure 1.

**Figure 2.**
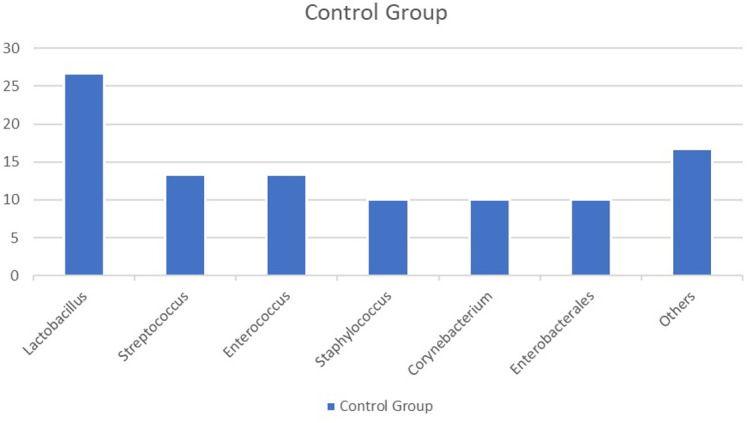
Microorganism distribution in the control group.

The most common bacterial species in the control group was *Lactobacillus gasseri* (n = 6, 20%) followed by *E. faecalis* (n = 4, 13.3%) and *Escherichia coli* (n = 3, 10%). The anaerobic bacteria, *Actinomyces neui*,* Finegoldia magna*, and *Mobiluncus mulieris* were identified in the control group. The distributions of the microorganisms identified in the control group are shown in Table 1 and Figure 2.

The frequency of bacterial species in the patient and control groups was examined with the Chi-square/Fisher’s exact test to determine any significant difference. A statistically significantly higher rate of *S. epidermis* was determined in the patient group (22.9%) than in the control group (0%) (*P* = .006). A statistically significantly lower rate of *L. gasseri* was determined in the patient group (2.9%) than in the control group (20.0%) (*P* = .042).

## Discussion

Overactive bladder is a syndrome in which there is a sudden feeling of urgency, generally accompanied by increased urine frequency and nocturia, and sometimes by urinary incontinence.^1^ Just as there is no gold standard method for the diagnosis of OAB, the etiology has not been fully clarified. It is thought that revealing the presence of urinary microbiota could have a place in OAB etiology.^2^,^4^

The leading study of patients with lower urinary symptoms was first conducted by Wolfe et al^2^ in 2012. This study showed that urine could be collected with a transurethral catheter in the investigation of urinary microbiota. That was the first study to reveal the presence of a microbiome in the bladder. The most frequently determined bacterial genera in that study were Lactobacillus, Aerococcus, Actinobaculum, Prevotella, Staphylococcus, Streptococcus, and Gardnerella. The greatest limitation of the study by Wolfe et al^2^ was that it could not be determined whether or not the bacteria identified belonged to the living microbiota. Therefore, for this purpose, the same team formed by Hilt et al^7^ in 2014 obtained urine samples with a transurethral catheter from 41 OAB patients and 24 control group subjects. In addition to 16S rRNA sequence analysis in these samples, inoculations were made with the EQUC method, which they had developed. The first of 2 important features of this study is that it provided evidence of the presence of live bacteria in the bladder. The second was that a correlation was shown between the 16S rRNA sequence analysis results and the EQUC method results. This demonstrates that this culture method could be used in the investigation of bladder microbiota. In that study, the most frequently determined bacterial genera were Lactobacillus, Corynebacterium, and Streptococcus. Of these, the most frequently determined species were *L. gasseri*,* Corynebacterium coyleae*, and *S. anginosus*. In the OAB patients, the most frequently determined bacterial genus was Corynebacterium. In the our study, the bacterial genera most frequently determined in OAB patients were Lactobacillus, Staphylococcus, and Streptococcus, and the most frequently determined species were *S. epidermidis* and *S. anginosus*. In the control group, the bacterial genera most frequently determined were Lactobacillus, Streptococcus, and Enterococcus, and the most frequently determined species were *L. gasseri* (20%) and *E. faecalis* (13.3%). The Facklamia and Finegoldia genus bacteria determined in the patient group by Hilt et al^7^ were determined in the control group of the our study.

One of the reasons for the significant differences between our study and this study may be that the culture methods we used were different. Another reason may be that the participants had different ethnic origins and geographic locations. The bladder microbiota may be affected by ethnic origin and geographic locations, similar to the gastrointestinal system.^9^ Multicenter studies are needed to demonstrate this.

In a 2014 study by Pearce et al^10^, bladder microbiota was compared in female patients with urgency type urinary incontinence (n = 23) and a control group (n = 25). In that study, 9 bacteria genuses (Actinobaculum, Actinomyces, Aerococcus, Arthrobacter, Corynebacterium, Gardnerella, Oligella, Staphylococcus, Streptococcus) were determined more frequently in the patient group than in the control group. Lactobacillus genus bacteria were determined at similar frequencies in both groups, but there were differences at the species level. *Lactobacillus gasseri* species was determined more frequently in the patient group, and *L. crispatus* more frequently in the control group. An important finding from that study was that significant differences were determined between the bladder microbiota of the patients and the control group. This suggests that there could be significant effects of this in the treatment and prevention of the disease. In the our study,* S. epidermis* was determined at a significantly higher rate in the OAB patient group (22.9%, *P* : .006) than in the control group. Unlike the study by Pearce et al^10^, *L. gasseri* was determined at a significantly higher rate in the control group (20%, *P *: .042) of our study compared to the patient group. While Pearce et al^10^ determined Finegoldia and Facklamia genus bacteria in the patient group of their study, these were only determined in the control group of the our study. The studies we mentioned were conducted in the same hospital in the USA. The similar results may be due to participants living in the same location. Our study was conducted in Türkiye with participants of Turkish origin. This may be the reason for the significant differences between these studies.

A 2016 study by Karsten et al^11^ included 10 patients with urgency type urinary incontinence and a control group of 9 individuals. Urine samples were collected with a transurethral catheter, and the presence of bacteria was determined with 16S rRNA sequence analysis. There was growth in all urine samples taken. They found 190 bacterial species in the patient group and 153 bacterial species in the control group. In the study by Hilt et al^7^, there was growth in 80% of the urine samples and 35 different bacterial species were detected. In the study by Pearce et al^10^ (2014), growth occurred in 64.8% of the urine samples and 386 different bacterial species were detected. In our study, bacteria were detected in 20 (100%) of 20 patient samples and 17 (85%) of 20 control samples. A total of 16 different bacteria genuses and 29 different species were determined. This difference could be attributed to the different methods used in the studies. As in our study, Lactobacillus was the most frequently determined bacterial genus in the study by Karsten et al.^11^ In contrast to our study, Karsten et al^11^ found no significant difference between the patient and control groups.

In a study conducted in China by Wu et al^12^ in 2017, urine samples were collected via transurethral catheter from 30 OAB patients and 25 control individuals. From the total 55 samples, 689 bacterial genera were determined. Sneathia, Staphylococcus, Proteus, Helcococcus, Gemella, Mycoplasma, and Aerococcus were found significantly more in the group with OAB. Lactobacillus was determined at the rate of 30.2% in the patient group and at 41.2% in the control group, whereas in our study these rates were 26.7% in the control group and at the lower rate of 14.3% in the patient group, but in both studies, the difference was not statistically significant. Although there was no statistically significant difference, the number of bacteria (n = 35) determined in our study patient group and the variety of bacteria species (n = 21) were higher than in the control group (n = 30, n = 16). This result is different from that of several previous studies, which could be attributed to the different methods used or the different ethnic origins of the study participants.^9^

In the our study, Lactobacillus was determined at a lower rate in the patient group (14.3%) than in the control group (26.7%), which was similar to the findings of previous studies. The predominant species determined in the control group was *L. gasseri*. Lactobacillus genus members disrupt adenosine triphosphate production and the flow of Ca2+ secreted by uropathogenic bacteria, thereby preventing contraction of the bladder detrusor muscle.^13^ The presence of Lactobacillus genus bacteria in the urinary microbiota suggests that there is a protective effect against OAB with this and other as yet unclarified mechanisms.^10^,^12^

In the our study, a modified form of the EQUC method developed by Hilt et al^7^ was used, and inoculating was made in anaerobic blood culture bottles. Of the total 40 urine samples taken, production was determined in 4 (10%) only in the inoculation made in the anaerobic blood culture bottles. Therefore, it can be considered that including anaerobic blood culture bottles in the EQUC method could increase the rate of determination of bacteria in the microbiota. This is an important development as it contributes to culture-based bladder microbiota research.

There were some limitations in our study. One of them was the small sample size, and the other was the wide age range. Due to some financial and time problems, it was not possible to increase the sample size and keep the age range narrower.

In conclusion, our study shows that patients with OAB have a significantly different microbiota compared to the control group. More work is needed to develop culture-based microbiota methods and to investigate whether changing microbiota in diseases is a cause or a result.

## Figures and Tables

**Table 1.** Microorganisms Growing at Different Steps in the Modified Expanded Quantitative Urine Culture of Each Participant. Steps 1-5 Are Described in the Material and Methods Section

	Step 1	Step 2	Step 3	Step 4	Step 5 (Anaerobic)	Step 5 (Aerobic)
Patient 1				*S. anginosus*		*S. anginosus*
Patient 2				*S. epidermidis*	*L. rhamnosus*	*S. epidermidis*
Patient 3	*S. epidermidis*	*S. epidermidis*	*A. urogenitalis*	*S. epidermidis*	*A. urogenitalis*	*S. epidermidis*
Patient 4						*S. epidermidis* + *E. faecium*
Patient 5		*E. clocae*		*E. clocae*		*E. clocae*
Patient 6		*E. faecalis*	*L. gasseri*	*E. faecalis*	*L. gasseri*	*E. faecalis*
Patient 7	*S. epidermidis*	*S. epidermidis*		*S. epidermidis*		*S. epidermidis*
Patient 8	*C. glabrata*	*C. glabrata*		*C. glabrata*	*S. epidermidis*	*S. epidermidis*
Patient 9	*S. anginosus*	*S. anginosus*		*S. anginosus*		*S. anginosus*
Patient 10	*S. epidermidis + C. albicans*	*S. epidermidis* + *C. albicans*		*S. epidermidis* + *C. albicans*		*S. epidermidis* + *C. albicans*
Patient 11	*A. haemolyticum*					
Patient 12						*E. faecalis*
Patient 13	*A. haemolyticum*	*A. haemolyticum* + *C. glabrata*		*A. haemolyticum* + *C. glabrata*	*L. fermentum*	*A. haemolyticum*
Patient 14					*L. jensenii*	
Patient 15	*K. pneumoniae*	*K. pneumoniae*		*K. pneumoniae*		*K. pneumoniae*
Patient 16		*E. faecalis*		*E. faecalis*		*E. faecalis*
Patient 17	*S. mitis* + *S. salivarus*	*S. mitis +S. salivarus* + *E. coli*		*S. mitis + S. salivarus + E. coli*		*S. mitis + S. salivarus + E. coli*
Patient 18	*S. epidermidis*	*S. epidermidis*		*S. epidermidis*		*S. epidermidis*
Patient 19	*S. anginosus + C. turbata*	*S. anginosus + C. turbata*	*L. crispatus*	*S. anginosus*		*E. coli*
Patient 20						*S. anginosus + S. epidermidis*
Control 1						
Control 2	*S. salivarus*	*S. salivarus*	*L. jensenii*	*S. salivarus*	*L. jensenii*	*S. salivarus*
Control 3	*C. pseudotuberculosis*					*F. hominis*
Control 4						
Control 5						
Control 6	*L. jensenii + B. cepacia*	*B. cepacia + S. agalactiae*		*B. cepacia + S. agalactiae*		*B. cepacia + S. agalactiae*
Control 7	*E. faecalis*	*E. faecalis*		*E. faecalis*		*E. faecalis*
Control 8	*S. anginosus* + *C. amycolatum*	*S. anginosus*		*S. anginosus*		*S. anginosus*
Control 9			*L. gasseri*		*L. gasseri + A. neui*	*S. hominis*
Control 10						
Control 11		*L. gasseri*	*L. gasseri*		*L. gasseri*	*L. gasseri*
Control 12	*E. faecalis*		*F. magna*	*E. faecalis*		*E. faecalis*
Control 13			*L. gasseri + M. mulieris*		*L. gasseri*	
Control 14			*L. gasseri*			*E. faecalis*
Control 15		*L. gasseri*	*L. gasseri*			*E. faecalis*
Control 16	*S. haemolyticus*	*S. haemolyticus*				*E. coli*
Control 17		*E. coli*		*E. coli*		*E. coli*
Control 18	*L. gasseri*	*L. gasseri*	*L. gasseri*		*L. gasseri*	
Control 19	*C. amycolatum*	*S. haemolyticus*			*S. haemolyticus*	*E. coli*
Control 20		*S. anginosus*		*S. anginosus*		*S. anginosus*

**Table 2.** Distribution of Microorganism Genus in the Control and Patient Groups

	Patient		Control		
	Frequency	%	Frequency	%	*P*
Streptococcus*	6	17.1	4	13.3	.742
Staphylococcus**	9	25.7	3	10.0	.104
Enterococcus*	4	11.4	4	13.3	1.000
Corynebacterium*	0	0.0	3	10.0	.093
Arcanobacterium*	2	5.7	0	0.0	.495
Lactobacillus**	5	14.3	8	26.7	.213
Actinomyces*	1	2.9	1	3.3	1.000
Enterobacterales*a	4	11.4	3	10.0	1.000
Burkholderia*	0	0.0	1	3.3	.462
Candida*	3	8.6	0	0.0	.243
Facklamia*	0	0.0	1	3.3	.462
Finegoldia*	0	0.0	1	3.3	.462
Mobiluncus*	0	0.0	1	3.3	.462
Cellulomonas*	1	2.9	0	0.0	1.000

a, *Enterobacterales* includes the genera *Escherichia*, *Enterobacter*, and *Klebsiella.*

*Fisher’s exact test. **Chi-square analysis.

**Table 3.** Distribution of Microorganisms Species in the Patient and Control Groups

	Patient		Control		*P*
	Frequency	%	Frequency	%	
S. anginosus*	4	11.4	2	6.7	.678
S. salivarus*	1	2.9	1	3.3	1.000
S. mitis*	1	2.9	0	0	1.000
S. agalactiae*	0	0	1	3.3	.462
S. epidermidis*	8	22.9	0	0	3**.006**
S. haemolyticus*	1	2.9	2	6.7	.591
S. hominis*	0	0	1	3.3	.462
E. faecalis*	3	8.6	4	13.3	.695
E. faceium*	1	2.9	0	0	1.000
C. pseudotuberculosis*	0	0	1	3.3	.462
C. amycolatum*	0	0	2	6.7	.209
A. haemolyticum*	2	5.7	0	0	.495
L. jensenii*	1	2.9	2	6.7	.591
L. gasseri*	1	2.9	6	20.0	3**.042**
L. rhamnosus*	1	2.9	0	0	1.000
L. crispatus*	1	2.9	0	0	1.000
L. fermentum*	1	2.9	0	0	1.000
A. neui*	0	0	1	3.3	.462
A. urogenitalis*	1	2.9	0	0	1.000
E. coli*	2	5.7	3	10.0	.655
E. cloacae*	1	2.9	0	0	1.000
K. pneumoniae*	1	2.9	0	0	1.000
B. cepacia*	0	0	1	3.3	.462
C. albicans*	1	2.9	0	0	1.000
C. glabrata*	2	5.7	0	0	.495
Facklamia hominis*	0	0	1	3.3	.462
Finegoldia magna*	0	0	1	3.3	.462
Mobiluncus mulieris*	0	0	1	3.3	.462
Cellulomonas turbata*	1	2.9	0	0	1.000
Total	35	100	30	100	

*Fisher’s exact test.

## Data Availability

The data of this study is available upon request to the corresponding author.
